# *c-erbB2/neu* gene and chromosome 17 analysis in breast cancer by FISH on archival cytological fine-needle aspirates

**DOI:** 10.1038/sj.bjc.6690387

**Published:** 1999-05-01

**Authors:** A Mezzelani, L Alasio, C Bartoli, M G Bonora, M A Pierotti, F Rilke, S Pilotti

**Affiliations:** Divisione di Anatomia e Istologia Patologica e Citopatologia, Divisione di Semeiotica Chirurgica e Chirurgia Ambulatoriale, Divisione di Oncologia Sperimentale A, Istituto Nazionale per lo Studio e la Cura dei Tumouri, Via G. Venezian 1, 20133 Milano, Italy

**Keywords:** breast carcinoma, FNA, *c-erbB2/neu*, FISH, immunocytochemistry, primary chemotherapy

## Abstract

The detection of specific genetic alterations in breast cancer is useful for diagnosing, predicting prognosis and planning preoperative treatment. *c-erbB2/neu* overexpression is usually detected by immunocytochemistry (ICC), although this technique is neither completely reproducible nor highly reliable, owing to specimen and methodologic variability and antibody sensitivity. Here, we combine two well-established techniques, fine-needle aspiration (FNA) and fluorescence in situ hybridization (FISH), to detect *c-erbB2/neu* amplification in patients candidate to primary chemotherapy and, in part, previously analysed for *c-erbB2/neu* overexpression. Sixty smears from FNA were used to simultaneously detect *c-erbB2/neu* and chromosome 17 centromere. FISH was successful in 58 cases and detected 24 amplified cases, three of which were negative by immunophenotyping, 28 negative cases, with evidence of two normal *c-erbB2/neu*/ signals, two cases with deletion of *c-erbB2/neu*, and four cases with polysomy, thus providing more reliable and informative results than ICC. This study underlines the advantages offered by the FNA and FISH combination which are two rapid, reliable, simple and informative techniques, to analyse one of the most important genetic markers for predicting prognosis and chemotherapy planning for breast carcinoma in particular in the light of the recently proposed trials of primary chemotherapy. © 1999 Cancer Research Campaign


British Journal of Cancer (1999) **80**(3/4), 519–525
© 1999 Cancer Research Campaign
Article no. bjoc.1998.0387

### *c-erbB2/neu* gene and chromosome 17 analysis in breast cancer by FISH on archival cytological fine-needle aspirates

**A Mezzelani[1], L Alasio[1], C Bartoli[2], MG Bonora[1], MA Pierotti[3], F Rilke and S Pilotti[1]**

[1]Divisione di Anatomia e Istologia Patologica e Citopatologia, [2]Divisione di Semeiotica Chirurgica e Chirurgia Ambulatoriale, [3]Divisione di Oncologia Sperimentale A, Istituto Nazionale per lo Studio e la Cura dei Tumouri, Via G. Venezian 1, 20133 Milano, Italy

**Summary** The detection of specific genetic alterations in breast cancer is useful for diagnosing, predicting prognosis and planning preoperative treatment. *c-erbB2/neu* overexpression is usually detected by immunocytochemistry (ICC), although this technique is neither completely reproducible nor highly reliable, owing to specimen and methodologic variability and antibody sensitivity. Here, we combine two well-established techniques, fine-needle aspiration (FNA) and fluorescence in situ hybridization (FISH), to detect *c-erbB2/neu* amplification in patients candidate to primary chemotherapy and, in part, previously analysed for *c-erbB2/neu* overexpression. Sixty smears from FNA were used to simultaneously detect *c-erbB2/neu* and chromosome 17 centromere. FISH was successful in 58 cases and detected 24 amplified cases, three of which were negative by immunophenotyping, 28 negative cases, with evidence of two normal *c-erbB2/neu/* signals, two cases with deletion of *c-erbB2/neu*, and four cases with polysomy, thus providing more reliable and informative results than ICC. This study underlines the advantages offered by the FNA and FISH combination which are two rapid, reliable, simple and informative techniques, to analyse one of the most important genetic markers for predicting prognosis and chemotherapy planning for breast carcinoma in particular in the light of the recently proposed trials of primary chemotherapy.

**Keywords:** breast carcinoma; FNA; *c-erbB2/neu*; FISH; immunocytochemistry; primary chemotherapy

Fine-needle aspiration (FNA) is a well-established method for diagnosing breast cancer which has recently been successfully combined with molecular and cytogenetic analysis (Ljung et al, 1994). *c-erbB2/neu* overexpression is usually detected by the immunocytochemical (ICC) technique, although the methodological and specimen variability, and differences in antibody sensitivity, make this technique not entirely reproducible and, therefore, not highly reliable (Troncone et al, 1993; Corkill et al, 1994; Press et al, 1994; Midulla et al, 1995). On the other hand, the quantification of *c-erbB2/neu* copy number with semi-quantitative polymerase chain reaction (PCR) (Lonn et al., 1996) does not allow the performance of a 'cell by cell' analysis and the evaluation of gene distribution. The feasibility of dual-colour fluorescence in situ hybridization (FISH) on cytological material from FNA (performed on thawed tissue samples using a 24-gauge needle) has already been described (Sauter et al, 1996). Similarly, one-colour FISH on archival cytological samples up to 94 days old has been successfully performed (Cajulis et al, 1996). We have recently started to perform FNA for *TP53* molecular analysis in a number of patients candidate to primary chemotherapy for breast cancer (Lavarino et al, 1998). In this paper, we further developed a cytogenetic approach applying dual-colour FISH analysis of *c-erbB2/neu* (Kallionemi et al, 1992) to material obtained from the same pass from the same patient series and to a new series. *c-erbB2/neu*, which is located on chromosome 17q11.2-q12 and chromosome 17 pericentromeric probes, were co-hybridized on fresh cytological smears and, when the latter were not available, on destained smears. A number of cases, of which fresh frozen material was available, were also analysed for *c-erbB2/neu* overexpression by means of conventional ICC technique. The purposes of this analysis were as follows: (i) to verify the feasibility of a molecular-cytogenetic analysis for *c-erbB2/neu* gene amplification on FNA material, (ii) to verify the possibility of using frozen and/or destained smears, and (iii) to compare molecular-cytogenetic and ICC results. The results show that dual-colour FISH may be successfully performed on each type of smear. The quantification and evaluation of spatial distribution and the heterogeneity level of *c-erbB2/neu* amplification among the nuclei represent an obvious advantage of FISH over ICC. Moreover, the higher sensitivity and reproducibility, as well as the evaluation of the chromosome copy number, make FISH unique for the assessment of *c-erbB2/neu* gene amplification on FNA material.

*Received 2 July 1998*
*Revised 12 November 1998*
*Accepted 25 November 1998*

*Correspondence to*: A Mezzelani

### MATERIALS AND METHODS

### Patients and tumours

Fifty-eight consecutive female patients (age range 25–71 years; mean 53 years) with primary breast carcinoma ascertained by FNA, who had entered a primary chemotherapy protocol with doxorubicin and paclitaxel, were selected for the present study. According to the tumour node and metastasis classification (TNM; UICC, 1992), one case was T4bN2M1, one case T4bN2M0, three cases were T4bN1M1, 12 cases T4bN1M0, one case T3N1M1, four cases T3N1M0, three cases T3N0M0, one case T2N2M0, 17 cases T2N1M0, 11 cases T2N0M0, three cases T1N1M0 and one case T1N0M0. Two patients came from breast cancer-prone families.

### FNA

In each case material was obtained by 10–15 rapid back and forth motions of the fine needle (21-gauge) within the tumour tissue nodule performed during a single pass. In one case, material from both the primary tumour and a nodal metastasis was available, and in another case from two nodules from both breasts.

### Cytological preparations

The aspirated material was partly smeared on slides; one slide was fixed immediately in 95% ethanol and stained according to the Papanicolaou method (PAP) (Koss, 1979*a*), one air-dried and stained with the May–Grünwald–Giemsa technique (MGG) (Koss, 1979*b*), for morphologic evaluation, and the other slides were frozen. The remaining material was resuspended in phospate-buffered saline (PBS) and frozen.

Additional information (data not shown) was acquired by an immunocytochemical panel, routinely applied to FNA material in our institution, encompassing hormonal receptors and proteins encoded by the *BCL2* and *TP53* genes. Assessment of estrogen receptor (ER) and progesterone receptor (PR) was carried out as previously described (Frigo et al, 1995), and of *BCL2* and p53 proteins by monoclonal antibody (mAb) bcl-2,124 (1:1000 diluted) (DAKO, Glostrup, Denmark), and mAb DO7 (1:1000 diluted) (Ylem, Avezzano, AQ, Italy), respectively, as detailed above.

### Immunostaining

Detection of *c-erbB2/neu* protein expression was performed on fresh smears using the mAb CB11 (1:1000 diluted) (Ylem, Avezzano, AQ, Italy). The antibody was detected with the streptavidin–biotin immunoperoxidase method (streptavidin HRP: horseradish peroxidase). Briefly, smears were treated with 0.3% hydrogen peroxide for 20–30 min to suppress endogenous peroxidase. Thereafter, 2% normal human serum was applied for 30 min as a suppressor serum. The slides were then incubated overnight at 4°C with the primary antibody. After several brief rinses, the biotinylated secondary antibody (30 min) and streptavidin HRP were applied in succession. The preparations were then developed in 3,3-diaminobenzidine solution, counterstained in Carazzi's haematoxylin, dehydrated and mounted. On cytological material *c-erbB2/neu* staining was interpreted as positive when the whole cell was decorated with a sharper signal associated with cytoplasmic boundaries.

### Specimens for FISH analysis

Sixty aspirates (one patient had one lymph node and the breast nodule aspirated, and one had one nodule for each breast) were analysed: three were frozen smears, 29 were cytospins from frozen material in PBS suspension, four were cytological smears stained with MGG and 24 were cytological smears stained with PAP.

### Pretreatment of slides

Cytological smears stained with PAP or MGG were destained and treated prior to in situ hybridization according to Cajulis and collaborators (1996).

### Dual-colour FISH

On each slide, an area of 18 × 18 $mm^2$ was hybridized with the biotinylated probe *c-erbB2/neu* (ERBB2) (Oncor, Gaithersburg, MD, USA) together with the digoxigenin-labelled chromosome 17 α-satellite region (D17Z1) (Oncor, Gaithersburg, MD, USA). The hybridization was performed according to Lichter (Lichter et al, 1990) and the manufacturer's recommendations.

The biotinylated probes *c-erbB2/neu* were detected by two layers of avidin–FITC (fluorescein isothiocyanate; Vector, Burlingame, CA, USA), and chromosome 17 α-satellite by one layer of rhodamine-labelled anti-digoxigenin (ab) (Boehringer Mannheim, Germany). Slides were then counterstained by DAPI (4,6-diamidino-2-phenylindole dihydrochloride hydrate).

#### *FISH results evaluation*

The slides were observed at 1000× magnification. At least 100 well-defined nuclei were scored for each hybridization. Samples were considered:

*Normal:* when two *c-erbB2/neu* related to two chromosome 17 copy number was found in all cells.
*Amplified:* when *c-erbB2/neu* copy number per centromere 17 is greater than 2.
*Polysomy:* when equal numbers of *c-erbB2/neu* and centromere 17 signals are present and the numbers are greater than 2.
*Deleted:* when *c-erbB2/neu* are fewer than centromere 17 copies.

#### *Digital signal detection*

Image acquisition was performed with a cooled CCD camera (Photometrics, Tucson, AZ, USA) coupled with a Zeiss Axioskop fluorescence microscope and controlled by a Power Macintosh 7100/80. Frames of the nuclei were taken separately using the software package IPLab Spectrum (Signal Analytics).

### RESULTS

The results obtained are summarized in Table 1. Both frozen and PAP or MGG stained cytological smears were suitable for FISH analysis. *c-erbB2/neu* probe successfully hybridized 58 out of 60 samples (96%). On the other hand, chromosome 17 pericentromeric signals were detectable in 31 out of 60 (51%) slides, most probably due to technical problems: in fact *c-erbB2/neu* was detected with two layers of avidin–FITC while chromosome 17 α-satellite was detected with only one layer of rhodamine-labelled anti-digoxigenin antibody (so as to avoid background artefacts). No differences were observed between PAP and MGG colouring and the different types of slide preparations.

Considering the clinically significant results we observed the following:

#### c-erbB2/neu amplification

Twenty-four cases (36.6%) were amplified. All of them showed a clustered distribution, although a small number of cases also

 *© Cancer Research Campaign 1999*

**Table 1** Results obtained by FISH analysis on 58 of 60 cases stored frozen and destained smears, of *c-erbB2/neu* related to chromosome 17 copy number

| | | No. of cases with: | | | |
|---|---|---|---|---|---|
| ***c-erbB2/neu*** | **No. of cases (%)** | **Normal chromosome 17** | **Monosomic chromosome 17** | **Polysomic chromosome 17** | **Chromosome 17 not evaluable** |
| Amplified | 24 (41.4) | 15 | 1 | 4 | 14 |
| Normal | 28 (48.3) | 5 | 0 | 0 | 13 |
| Deleted | 2 (3.4) | 0 | 1 | 1 | 0 |
| Polysomic | 4 (6.9) | 0 | 0 | 4 | 0 |
| Total | 58 | 20 | 2 | 9 | 27 |

showed a few diffuse signals. As the level of gene amplification was heterogeneous among the nuclei of the same specimen, an average was evaluated and the cases were then subclassified on the basis of the results obtained.

*Very low level of amplification*: only one case, with 4–5 *c-erbB2/neu* signals out of two chromosome 17, presented a very low level of gene gain in 10% of the cells (Figure 1 C,D)
*Low level of amplification* (from 6 to 15 signals): seven cases were found
*Medium level of amplification* (from 16 to 30): ten cases showed this grade of gene amplification.
*High level of amplification* (more than 30 signals): only four cases showed this *c-erbB2/neu* amplification.

Moreover, five cases revealed two normal chromosome 17, four cases (which resulted aneuploid by ploidy analysis), (Lavarino et al, 1998), showed polysomy of chromosome 17, one case had only one chromosome 17 and the remaining eight were not available for this parameter.

### *Normal* c-erbB2/neu

Twenty-eight cases (43.3%) showed two *c-erbB2/neu* signals. In 15 of them the signals were related to two chromosome 17, and in the remaining 13 cases the chromosome 17 was not detectable.

### c-erbB2/neu deletion

Two cases (6.6%) showed *c-erbB2/neu* deletion. The first one had one *c-erbB2/neu* signal related to a monosomy of chromosome 17, and the second had two *c-erbB2/neu* signals out of three chromosome 17.

### c-erbB2/neu polisomy

Four cases (6.9%) showed polysomy of the gene linked to the polysomy of chromosome 17. The level of polysomy among the four cases ranged from trisomy up to 7–8 chromosome 17 and was related to an equal number of *c-erbB2/neu* copies.

**Table 2** Results obtained with ICC and FISH in 47 cases

| | *c-erbB2/neu* FISH | |
|---|---|---|
| **ICC** | **Amplified** | **Non-amplified** |
| Overexpressed | 17 | 0 |
| Non-overexpressed | 3 | 27 |
| Total | 20 | 27 |

All samples but one (see *Very low level of amplification*), scored as amplified, polysomic or deleted showed the genetic anomalies in at least 20% of the cells. All samples with gene amplification, polysomy or monosomy exhibited a high level of heterogeneity among individual cells. In spite of fine-needle aspirates, cells directly from the node, a contamination of blood cells or healthy tissue cells is frequent making cell population heterogeneous. On the other hand, in case of amplification, the variability of gene copy number among the tumour cells of the same specimens reflect genetic instability and may be an important characteristic of solid tumours since genetic instability is essential for tumour evolution (Sauter et al, 1996).

### *Comparison between FISH and ICC (Table 2)*

ICC analysis was carried out in 47 cases with available frozen smears and/or cytospin preparation. FISH results were concordant with previous ICC estimates in 44 cases. Three cases, negative for overexpression, appeared to be amplified by FISH. One case showed a very low amplification (consisting in 4–5 *c-erbB2/neu* signals out of two chromosome 17). As for the other two cases, FISH results showed a high level of amplification in contrast with the negative overexpression.

### DISCUSSION

Primary chemotherapy alone, or combined with radiotherapy, is being progressively widely applied to reduce mutilating surgery in the treatment of patients with breast carcinoma (Bonadonna et al, 1990; Rilke et al, 1996). Breast carcinomas that overexpress *c-erbB2/neu* are more aggressive (De Potter et al, 1995), less responsive to CMF-containing adjuvant therapy regimens (Gusterson et al, 1992), may benefit from high-dose adjuvant chemotherapy (Muss et al, 1994) and, when steroid receptor positive (Pietras et al, 1995), may be unresponsive to tamoxifen (Hynes et al, 1994) or to tamoxifen with radiotherapy (Sjogren et al, 1998). Moreover, Press and collaborators (1997), have demonstrated that *c-erB-2/neu* is an independent predictor of poor clinical outcome and, more recently, the Toronto Breast Cancer Study Group has found that *c-erbB2/neu* amplification is an independent prognostic factor for risk of recurrence in axillary node negative breast cancer (Andrulis et al, 1998). Recent clinical studies, in keeping with preclinical evidence (Harwerth et al, 1993; Baselga et al, *1996)*, have shown some benefits in stage IV *c-erbB2/neu* overexpressing breast carcinomas treated by recombinant humanized monoclonal (rhmAb) *c-erbB2/neu* (Baselga et al, 1996). In this scenario, reliable information on *c-erbB2/neu* overexpression, in addition to other biological markers, becomes determinant in both predicting prognosis and planning treatment.

*© Cancer Research Campaign 1999*

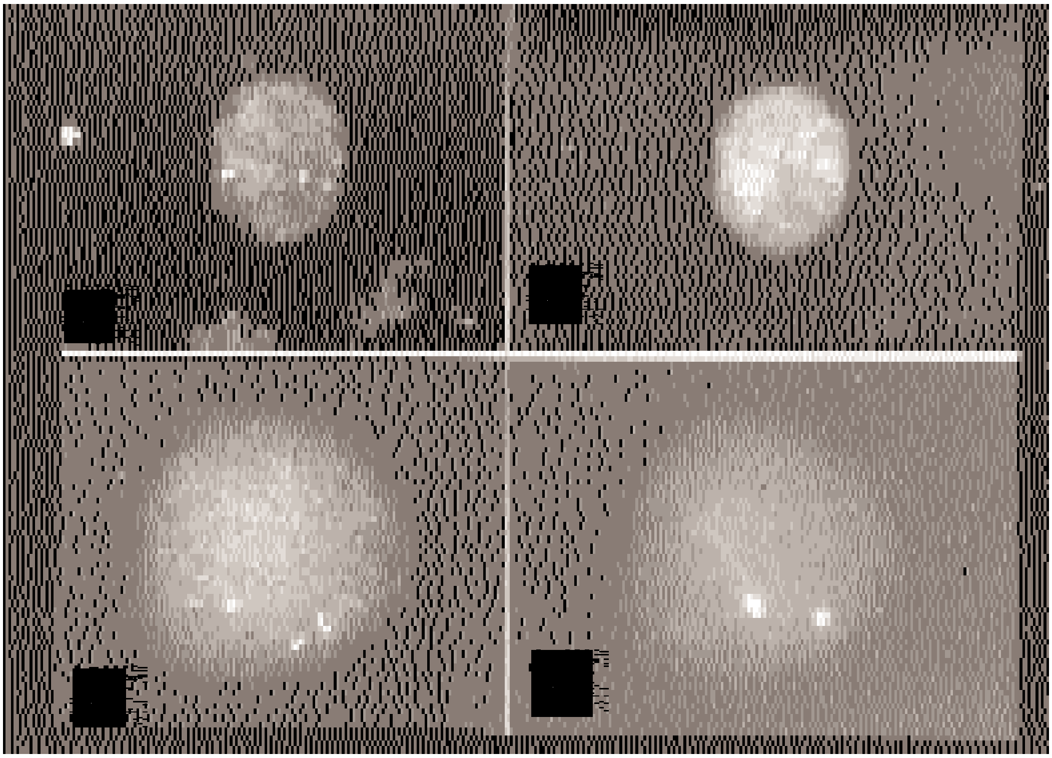

**Figure 1** (**A**) A nucleus with two normal *c-erbB2/neu* signals related to two normal chromosome 17 copy number shown in (**B**). (**C**) A nucleus with a very low level of gene amplification (5 spots) out of two chromosomes 17 shown in (**D**)

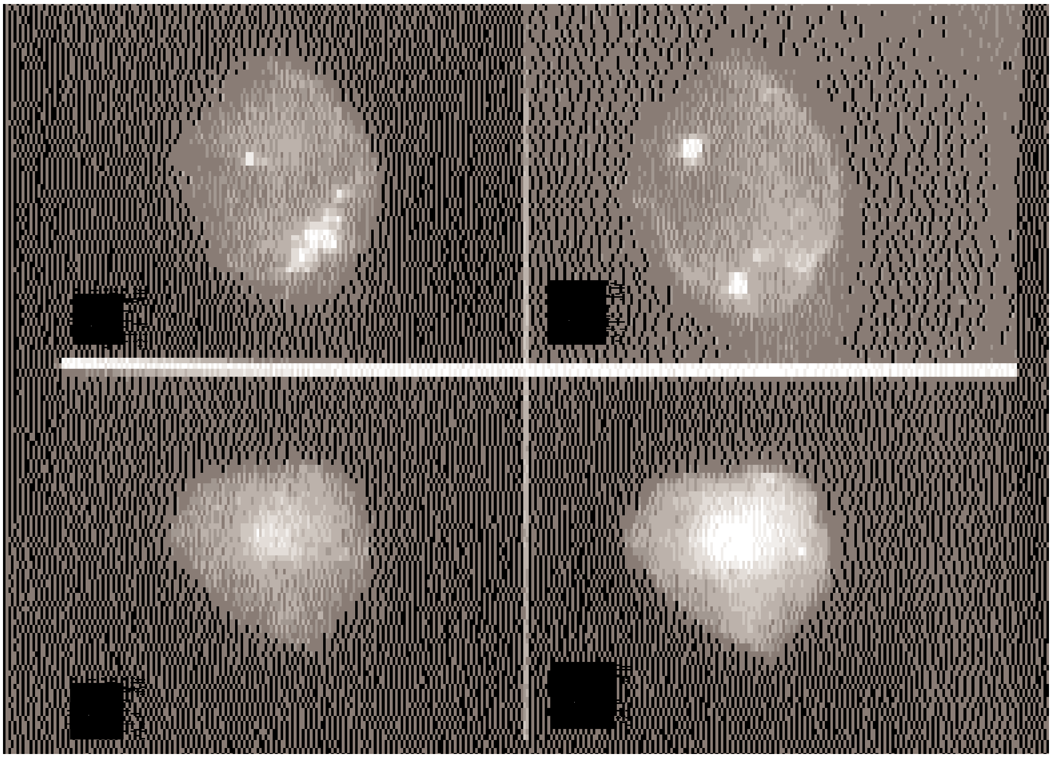

**Figure 2** (**A**) A nucleus showing a *c-erbB2/neu* amplification; it is noticeable that amplification has happened only in one of the two chromosome 17 shown in (**B**), as the other one has a normal single copy of the gene. (**C**) A case of *c-erbB2/neu* deletion that is related to a chromosome 17 deletion shown in (**D**)

*© Cancer Research Campaign 1999*

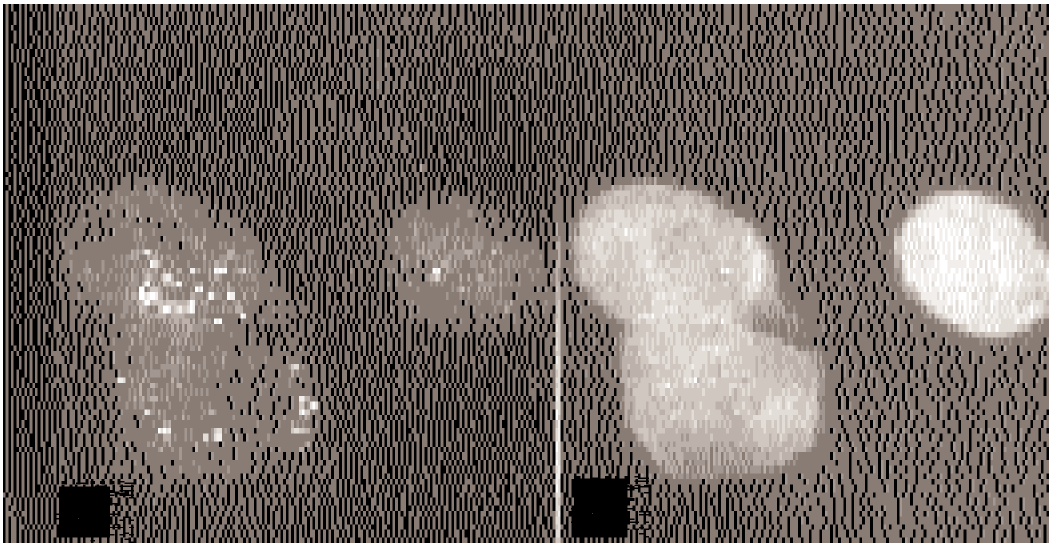

**Figure 3** (**A**) A case of *c-erbB2/neu* amplification; two nuclei have gene amplification as one has two normal copies. The same counterstained nuclei are shown in (**B**)

In our experience, the selective plasma membrane immunoreactivity of c-erbB2/neu protein shows a poor reproducibility on fresh or cryopreserved FNA material. Therefore, this gene product is not currently used for our pretreatment immunophenotyping panel routinely applied to patients meeting the criteria for primary chemotherapy, and which includes the assessment of hormonal receptors, the MIB1 growth fraction, and the proteins encoded by *TP53* and *BCL2* genes. Furthermore, the literature data on *c-erbB2/neu* immunoreactivity applied to cytology are contradictory; in fact, even though a higher cytological sensitivity is shown over histology (Corkill et al, 1994; Troncone et al, 1996), some authors claim that immunostaining performed on destained smears (Corkill et al, 1994;) are more informative, while others support fresh material immunostaining (Troncone et al, 1996). More worrying, the data on *c-erbB2/neu* overexpression in these cytology-based studies appear to largerly exceed (Troncone et al, 1993; Midulla et al, 1995) those based on molecular-histologic investigations, which yield a positive rate of 10–40% of cases examined (De Potter et al, 1995; Rilke et al, 1991). Such inconsistencies in antibody sensitivity and possible cross-reacting artefacts (Rilke et al, 1991; Press et al, 1994; Bobrow et al, 1996), prompted us to identify a more reliable and independent technique to complement the immunocytochemical panel data.

Our results show a fairly good correlation between *c-erbB2/neu* overexpression and gene amplification: all cases with positive immunostaining were amplified. In our study, however, three immunophenotypically negative cases, showed gene amplification by FISH: one showed a very low amplification in only 10% of the nuclei, which probably cannot be detected by the antibody while the second had a high degree of gene amplification which prompted us to re-examine the case that, following respect immunostaining, proved positive. The third was one of the two breast nodules of the same patients: both nodules showed a high level of amplification by FISH, but only one was positive by ICC. These results confirm the reliability of FISH analysis. Moreover, a negative FISH, compared to conventional ICC, has the advantage of presenting ‘two signals’ (or only one in case of deletion), that discriminate between a true negative result and the failure of the hybridization procedure. Moreover, as *c-erbB2/neu* amplification has prognostic significance, the possibility to quantify the level of gene amplification could better correlate in predicting prognosis.

FISH also allows the determination of spatial distribution of amplified genes, distinguishing between clustered and diffuse types of distribution. The first is suggestive of an intrachromosomal location of the gene copies, whereas the second is suggestive of an extrachromosomal location in double minutes (DM) (Kallionemi et al, 1992). Since preclinical evidence has shown that a low concentration hydroxyurea-based treatmentmay reduce extrachromosomic amplifications and tumour cell tumorigenicity, we could therefore speculate that DMs should represent an important target for chemotherapy (Von Hoff et al, 1992).

Another advantage of dual-colour FISH analysis is that the chromosome 17 copy number is simultaneously characterized, adding some prognostic information. As for chromosome 17 polysomy, it has been demonstrated that polysomy identified by FISH is related to DNA aneuploidy which in turn, in breast carcinoma, is related to a poor prognosis (Gnant et al, 1993). In fact, all our cases that were polysomic by FISH were aneuploid by static analysis of DNA content (Lavarino et al, 1998). Furthermore, in our (data not shown) and in another experience (Kallionemi et al, 1992), *c-erbB2/neu* overexpression may occur in association with polysomy. The significance of this type of overexpression has not been investigated in a clinical setting.

In the case of deletion of chromosome 17, there is the loss of several tumour suppressor genes located on this chromosome, including nm23, the familial breast cancer gene *BRCA1*, and *TP53*. Tumour suppressor genes are frequently inactivated by the deletion of one allele and the mutation of those remaining. Hence, tumours with a chromosome 17 monosomy present a higher risk of being, or of becoming carriers of homozygous inactivated forms of the above mentioned genes (Solomon et al, 1991), in particular *TP53*. For this reason, the cases analysed here are also currently being investigated for *TP53* mutational status (Lavarino et al, 1998). The rationale for the definition of these genetic alterations derived not only from preclinical (Lowe et al, 1993, 1994) and

© Cancer Research Campaign 1999

clinical (Wahl et al, 1996) evidence suggesting a high correlation between chemotherapy response and *TP53* status (Moll et al, 1995), but also from recent in vitro observations, relevant to the present findings, suggesting that *c-erbB2/neu* antibody-induced growth arrest of cancer cells depends on the activity of wild-type of *TP53* (Hansen et al, 1995). If a similar phenomenon happens in vivo in breast cancer patients, *TP53* status will become determinant for the prediction of the therapeutic response to a treatment with anti-*c-erbB2/neu* mAb.

In conclusion, our results underline the advantages offered by the combination of two rapid and very informative techniques, such as FNA and FISH, in a clinical setting in order to acquire additional data on genetic markers useful for the prediction of prognosis, for the planning of more individualized treatments and for the evaluation of therapy response (Goldhirsch et al, 1995).

### ACKNOWLEDGEMENTS

This work was supported by grants from AIRC/FIRC (Associazione and Fondazione Italiana per la Ricerca sul Cancro), and partially by CNR (Consiglio Nazionale delle Ricerche), PF 'ACRO'.

### REFERENCES

Alsabeh R, Wilson CS, Ahn CW, Vasef MA and Battifora H (1996) Expression of *bcl-2* by breast cancer: a possible diagnostic application. *Modern Pathol* **9**: 439–444

Andrulis IL, Bull SB, Blackstein ME, Sutherland D, Mak C, Sidloffsky S, Pritzker KPH, Hartwick RW, Hanna W, Lickley L, Wilkinson R, Qizilbash A, Ambus U, Lipa M, Weizel H, Katz A, Baida M, Mariz S, Stoik G, Dacamara P, Strongitham D, Geddie W and McCready D (1998) *neu/erB-2* amplification identifies a poor-prognosis group of women with node-negative breast cancer. *J Clin Oncol* **16**: 1340–1349

Baselga J, Tripathy D, Mendelsohn J, Baugham S, Benz CC, Dantis L, Sklarin NT, Bobrow LG, Happerfield LC and Millis RR (1996) Comparison of immunohistological staining with different antibodies to the c-erbB-2 oncoprotein. *Appl Immunohistochem* **4**: 128–134

Bonadonna G, Veronesi U, Brambilla C, Ferrari L, Luini A, Greco M, Bartoli C, Coopmans de Yoldi G, Zucali L, Rilke F, Andreola S, Silvestrini R, Di Fronzo G and Valugussa G (1990) Primary chemotherapy to avoid mastectomy in tumours with diameters of three centimeters or more. *J Natl Cancer Inst* **82**: 1548–1545

Cajulis RS, Frias-Hidvegi D, Gordon HY and Eggena S (1996) Detection of numerical chromosome abnormalities by fluorescence in situ hybridization of interphase cell nuclei with chromosome-specific probes on archival cytological samples. *Diagn Cytopathol* **14**: 178–181

Corkill ME and Katz R (1994) Immunocytochemical staining of c-erb B-2 oncogene in fine-needle aspirates of breast carcinoma: a comparison with tissue sections and other breast cancer prognostic factors. *Diagn Cytopathol* **11**: 250–254

De Potter CR and Schelfhout AM (1995) The *neu*-protein and breast cancer. *Virch Arch* **426**: 107–115

De Potter CR, Quatacker J, Maertens G, Van Daele S, Pauwels C, Verhofstede C, Eechaute W and Roels H (1989) The subcellular localization of the neu protein in human normal and neoplastic cells. *Int J Cancer* **44**: 969–974

Frigo B, Pilotti S, Zurrida S, Ermellino L, Manzari A and Rilke F (1995) Analysis of estrogen and progesterone receptors on preoperative fine needle aspirates. *Breast Cancer Res Treat* **33**: 179–184

Gnant M, Blijham G and Reiner A (1993) Aneuploid fraction but not DNA index is important for the prognosis of patients with stage I and II breast cancer: 10 year result. *Ann Oncol* **4**: 643–650

Goldhirsch A, Wood WC, Senn HJ, Glick JH and Gelber RD (1995) Meeting highlights: international consensus panel on the treatment of primary breast cancer. *J Natl Cancer Inst* **87**: 1441–1445

Gusterson BA, Gelber RD, Goldhirsch A, Price KN, Save-Soderborgh J, Anbazhagan R, Styles J, Rudenstam CM, Golouh R, Reed R, Martinez-Tello F, Tiltman A, Torhorst J, Grigolato P, Bettelheim R, Neville AM, Burki K, Castiglione M, Collins J, Lindtner J and Senn HJ (1992) Prognostic importance of c-erbB-2 expression in breast cancer. *J Clin Oncol* **10**: 1049–1056

Hansen R, Reddel R and Braithwaite A (1995) The transforming oncoproteins determine the mechanism by which p53 suppresses cell transformation: pRb-mediated growth arrest or apoptosis. *Oncogene* **11**: 2535–2545

Harwerth IM, Wels W, Schlegel J, Muller M and Hynes NE (1993) Monoclonal antibodies directed to the erbB-2 receptor inhibit in vivo tumour cell growth. *Br J Cancer* **68**: 1140–1145

Hynes NE and Stern DF (1994) The biology of *erbB-2/neu*/HER-2 and its role in cancer. *Biochim Biophys Acta* **1198**: 165–184

Kallionemi OP, Kallionemi A, Kurisu W, Thor A, Chen LC, Smith HS, Waldman FM, Pinkel D and Gray JW (1992) ERBB2 amplification in breast cancer analyzed by fluorescence in situ hybridization. *Proc Natl Acad Sci USA* **89**: 5321–5325

Koss LG (1979*a*) *Diagnostic Cytology and its Histopathologic Bases*, vol. 2, 3rd edn, pp. 1211–1222 Lippincott: Philadelphia–Toronto

Koss LG (1979*b*) *Diagnostic Cytology and its Histopathologic Bases*, vol. 2, 3rd edn, pp. 1006–1241. Lippincott: Philadelphia–Toronto

Lavarino C, Corletto V, Mezzelani A, Della Torre G, Bartoli C, Riva C, Pierotti MA, Rilke F and Pilotti S (1998) Detection of *TP53* mutation, loss of heterozygosity and DNA content in breast carcinoma fine-needle aspiration. *Br J Cancer* **77**: 125–130

Lichter P, Chang Tang C, Call K, Hermanson G, Evans GA, Housman D and Ward DC (1990) High-resolution mapping of human chromosome 11 by in situ hybridization with cosmid clones. *Science* **247**: 64–67

Ljung BM, Chew K, Deng G, Matsumura K, Waldman F and Smith H (1994) Fine needle aspiration techniques for the characterization of breast cancers. *Cancer* **74**: 1000–1005

Lonn U, Lonn S, Nilsson B and Stenkvist B (1996) Prognostic significance of c-erb-B2 amplification in fine-needle biopsies of breast cancer patients not operated at diagnosis. *Breast Cancer Res Treat* **39**: 213–220

Lowe SW, Ruley HE, Jacks T and Housman DE (1993) p53-dependent apoptosis modulates the cytotoxicity of anticancer agents. *Cell* **74**: 957–967

Lowe SW, Bodis S, McClatchey A, Remington L, Ruley HE, Fischer DE, Housman DE and Jacks T (1994) p53 status and efficacy of cancer therapy in vivo. *Science* **266**: 807–810

Midulla C, Giovagnoli MR, Valli C and Vecchione A (1995) Correlation between ploidy status ERB-B2 and P53 immunohistochemical expression in primary breast carcinoma. *Analyt Quant Cytol Histol* **17**: 157–162

Moll UM, Ostermeyer AG, Ahomadegbe JC, Mathieu MC and Riou G (1995) p53 mediated tumour cell response to chemotherapeutic DNA damage: a preliminary study in matched pairs of breast cancer biopsies. *Hum Pathol* **26**: 1293–1301

Muss HB, Thor AD, Berry DA, Kute T, Liu ET, Koerner F, Cirrincione CT, Budman DR, Wood WC, Barcos M and Handerson C (1994) c-erbB-2 expression and response to adjuvant therapy in women with node-positive early breast cancer. *N Engl J Med* **1330**: 1260–1266

Pietras RJ, Arboleda J, Reese DM, Wongvipat N, Pegram MD, Ramos L, Gorman CM, Parker MG, Sliwkowski MX and Slamon DJ (1995) *HER*-2 tyrosine kinase pathway targets estrogen receptor and promoters hormone-independent growth in human breast cancer cells. *Oncogene* **10**: 2435–2446

Press MF, Hung G, Godolphin W and Slamon DJ (1994) Sensitivity of c-erbB2/neu antibodies in archival tissue samples: potential source of error in immunohistochemical studies of oncogene expression. *Cancer Res* **54**: 2771–2777

Press MF, Bernstain L, Thomas PA, Meisner LF, Zhou JY, Ma Y, Hung G, Robinson RA, Harris C, El-Naggar A, Slamon DJ, Phillips RN, Ross JS, Wolman SR and Flom KJ (1997) HER-2/neu gene amplification characterized by fluorescence in situ hybridization: poor prognosis in node-negative breast carcinoma. *J Clin Oncol* **15**: 2894–2904

Rilke F, Colnaghi MI, Cascinelli N, Andreola S, Baldini MT, Bufalino R, Della Porta G, Menard S, Pierotti MA and Testori A (1991). Prognostic significance of *c-erbB2/neu* expression in breast cancer and its relationship to other prognostic factors. *Int J Cancer* **49**: 44–49

Rilke F, Veronesi U, Luini A, Brambilla C, Galimberti V, Zurrida S, Pilotti S, Di Palma S, Zucoli R and Lozza L (1996) Preoperative chemotherapy alone and combined with preoperative radiotherapy in small-size breast cancer. *Breast* **2**: 176–180

Sauter G, Feichter G, Torhorst J, Moch H, Novotna H, Wagner U, Durmuller U and Waldman FM (1996) Fluorescence in situ hybridization for detecting ErbB-2 amplification in breast tumour fine needle aspiration biopsies. *Acta Cytol* **40**: 164–173

Sjogren S, Inganas M, Lindgren A, Holmberg L and Bergh J (1998) Prognostic and predictive value of c-erB-2 overexpression in primary breast cancer, alone and in combination with other prognostic markers. *J Clin Oncol* **16**: 462–469

© Cancer Research Campaign 1999

Solomon E, Borrow J and Goddard A (1991) Chromosome aberrations and cancer. *Science* **254**: 1153–1160

Troncone G, Zeppa P, Fulciniti F, Di Benedetto G, Lombresco MA, De Divitiis B, Vetrani A and Palombini L (1993) c-erbB-2 expression and DNA ploidy status in breast cancer cells obtained by fine needle aspiration (FNA). *Cytopathology* **4**: 195–205

Troncone G, Panico L, Vetrani A, de Divitiis B, Zeppa P, Fulciniti F, Pettinato G and Palombini L (1996) c-erbB-2 expression in FNAB smears and matched surgical specimens of breast cancer. *Diagn Cytopathol* **14**: 135–139

Hermanek P and Sobin LH (eds) (1992) *TNM Classification of Malignant Tumours*, 4th edn, pp. 103–109. Springer Verlag: Berlin

Von Hoff DD, McGill JR, Forseth BJ, Davidson KK, Bradley TP, Van Devanter DR andWahl GM (1992) Elimination of extrachromosomally amplified MYC genes from human tumour cells reduces their tumorigenicity. *Proc Natl Acad Sci USA* **89**: 8165–8169

Wahl AF, Donaldson KL, Fairchild C, Lee FYF, Foster SA, Demers GW and Galloway DA (1996) Loss of normal p53 function confers sensitization to taxol by increasing G2/M arrest and apoptosis. *Nat Med* **2**: 72–79

*© Cancer Research Campaign 1999*
